# Emotional Creativity in Art Education: An Exploratory Analysis and Research Trends

**DOI:** 10.3390/ijerph18126209

**Published:** 2021-06-08

**Authors:** Mariana-Daniela González-Zamar, Emilio Abad-Segura

**Affiliations:** 1 Department of Education, University of Almeria, 04120 Almeria, Spain; 2 Department of Economics and Business, University of Almeria, 04120 Almeria, Spain

**Keywords:** emotional creativity, design, art education, wellbeing, scientific production

## Abstract

The emotions that human beings experience have a key role in the environments in which they operate. In art education, creative processes are influenced by the emotions and experiences lived by the individual, enabling a more emotional and creative design to make life more pleasant. The aim was to examine the research during the period 1917–2020 on the development of emotional creativity in art education. Mathematical and statistical techniques were applied to 984 articles carried from Elsevier’s Scopus database. The findings yielded data on the scientific productivity of the journal, authors, research institutions, and countries/territories that promoted this field. The data showed an exponential trend, mostly in the last decade. Five lines of research stand out: emotion, higher education, education, art, and leadership. Moreover, five future research directions related to visual art education, affective paradigm, metacompetency, expressive arts therapy group, and cognitive empathy were detected. This study establishes the link between psychology, neuroscience, and artistic education to constitute the decision-making of the promoters of this topic of research. The analysis of international research allowed us to focus the future publications of academics and researchers, in addition to guaranteeing an adequate approach to the objectives of the institutions and funding centers.

## 1. Introduction

The emotions experienced by human beings and the physiological and behavioral triggered reactions are key in the way of apprehending and participating in the environment that surrounds them [1,2]. In this set of circumstances, art education nurtures the creativity, creative thinking, and imagination of the student. Therefore, in educational centers, artistic thinking tends to enhance the imagination and creativity of the student, so that the arts help them develop expressiveness and the ability to connect with their environment [3,4].

The emotional creativity revealed in the educational stage assumes that the artistic skills acquired will act as a competitive advantage over those students who did not develop them during their academic process [5]. In the context of research, during the last few decades, there has been a growing number of studies that connect both emotions and creativity, creative and emotional competences, and design, highlighting their link with art education [6,7,8].

The purpose of the study was to detect and analyze the key elements of research on emotional creativity in arts education. The reviewed literature allowed us to raise the following research questions on emotional creativity in art education:

- What was the trend in research during the period analyzed?
- What was the relationship between the journals that developed this theme?
- What were the main scientific collaborations between researchers, countries/territories, and institutions?
- What were the main lines of research developed during the period 1917–2020?
- What are the main future research directions?

The objective was to analyze the research during the period 1917–2020 and determine the emerging directions of research on emotional creativity in arts education. To obtain answers to these issues, a sample of 984 articles chosen from the Scopus database was examined and mathematical and statistical techniques were applied. It is possible to determine through bibliometric indicators the growth of articles in this scientific field and to understand research trends.

The key contribution of this research has been to define the scientific literature and the cooperation networks between the developers of the theme of emotional creativity in art education. Moreover, the major lines of research developed to date and the most outstanding research orientations were identified. The analysis of international research allows us to focus the future publications of academics and researchers, in addition to guaranteeing an adequate approach to the objectives of the research institutions and funding centers.

To achieve the aim, the rest of this paper is organized as follows. Section 2 exposes the importance of the research topic, showing a review of its basic concepts. Section 3 describes the methodology applied to the sample of selected documents. Section 4 displays and discusses the findings in a comprehensive context. Section 5 contains the conclusions.

## 2. Theoretical Framework

The reviewed scientific literature has offered meanings of variables regarding emotional creativity in art education. The key terms used in this study are included to avoid confusion in their interpretations.

Emotions are subjective reactions to the environment that are accompanied by organic, physiological, and endocrine changes of innate origin [9] and appear suddenly in the form of violent and temporary crises [10]. Various studies indicate emotions as motivational systems with behavioral, experiential, and cognitive mechanisms, which (i) have a positive or negative valence; (ii) fluctuate in intensity; and (iii) are usually caused by personal situations, which involve attention by affecting well-being [11,12]. In any case, the study of emotion is complex, because it can undergo transformations, causing its conceptualization to generate a debate about the relevance of cognition and physiological foundations [13,14].

Artists and scientists from various disciplines consider the creative individual as a person with the ability to solve problems, develop products, and define new topics in each thematic field [15,16]. Hence, imagination, as the nucleus of creativity, allows us to think about aspects not perceived by the senses. This implies creating a teaching model that is based on creativity to prepare students for change, inviting them to learn to think [17,18]. Positive emotional states can boost creativity, allowing more ideas to be produced, although not necessarily more original; while negatives help people to produce more ideas when the creative task is considered interesting [19,20,21].

In these terms, emotional creativity is a basic extension of a social-constructionist view of emotion. The relationship between emotions and creativity is complex and fraught with ambivalence. Creativity is encouraged in schools, and in the arts and sciences, the highest praise for achievement is reserved. For these reasons, emotional creativity refers to a cognitive ability, and not only as the result of adding emotions and creativity. In this sense, various authors describe it as the ability to experience and express original, appropriate, and authentic combinations of emotions [22,23,24].

Likewise, emotional creativity may involve (i) the particularly effective application of an existing emotion or combinations of emotions (effectiveness); (ii) modifying a standard display to better meet the needs of the individual or group (authenticity); and (iii) the development of new forms of expression, with fundamental changes in the beliefs and rules by which emotional syndromes are constituted (novelty) [25,26,27]. Cultural variations presuppose emotional creativity at the individual level, suggesting a different way of looking at emotions and their disorders. This means that in a changing world, emotional creativity is not only an academic curiosity, but is anticipated as indispensable for social and psychological well-being [28,29].

In this field of research, the concept of art, as part of the tangible and intangible culture of a society, has a social role for its individuals [30]. Nowadays, the arts are a key component of the curriculum, fostering both creativity and self-expression and recognition of the expression of fellow students [31,32]. In this context, art education refers to the connection in artistic and creative activities [33], which encourages the development of artistic thinking to express ideas and emotions, in addition to interpreting the different keys of art [34,35]. Numerous studies have focused on the positive and necessary academic effects in an ever-evolving society that arts-based learning has on character acuity, motivation, increased interactions with peers, and conflict resolution skills [36,37].

## 3. Dataset and Methods

Statistical and mathematical techniques are utilized to suggest an approach to research related to emotional creativity in art education. The aim of this method is to detect and analyze the key elements within a certain field of research [38,39]. Likewise, it seeks to publicize the development of interest in the subject under analysis, reflecting the most prolific authors, countries/territories, research institutions, journals, and keywords of a specific period. Its use is based on the function that documents have in spreading new knowledge. Bibliometric indicators measure scientific productions and analyze the impact that works have on the scientific community [40]. The use of statistical data allows for descriptions related to science. These methods allow for the generation, visualization, and exploration of bibliographic maps, in order that the links and documents form a joint bibliographic network.

### 3.1. Data Extraction

An examination of the scientific information was carried out from the Scopus database. The search equation included the terms that reveal the scientific production of this research field: “education”, “art”, “artistic”, “design”, “creativity”, and “emotion”. These search terms were detected from a review of the literature (see Section 2).

**Figure 1:**
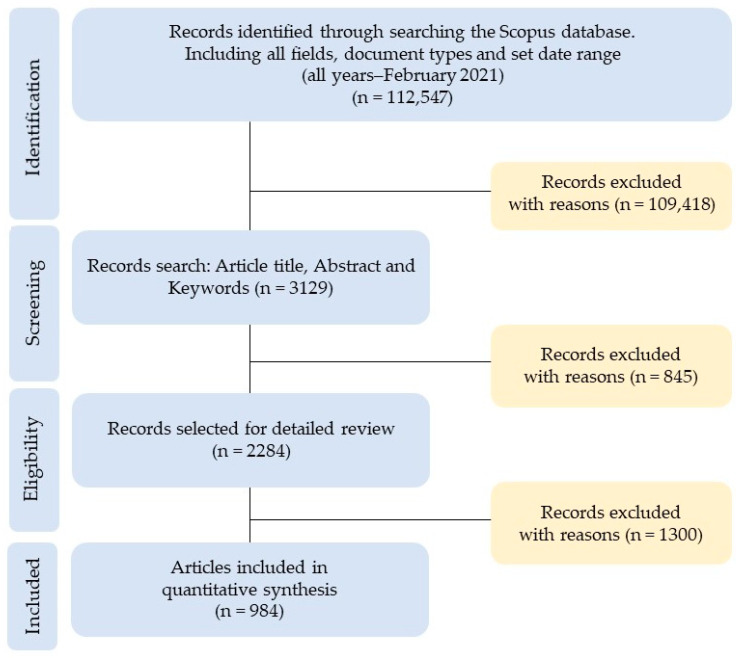
Selection of the sample of articles based on PRISMA.

Nowadays, there are no doubts about the advantages of using bibliographic databases, so the quality and validity of a research will depend to some extent on these. Some works have tried to answer the question about which database is more suitable for bibliometrics. Web of Science (WoS) and Scopus raise the question of the contrast and stability of the statistics obtained from the data sources. The evaluation of these databases has not clearly opted for one of them, as it will depend on both the scientific discipline and the period of analysis [41,42]. In this study, the choice of the Elsevier Scopus database was motivated because the data search showed a significant difference in the volume of articles in the analyzed period 1917–2020 between both databases, that is, WoS (150) and Scopus (984). The process for selecting the sample followed the flowchart in Figure 1, based on the Preferred Reporting Items for Systematic Reviews and Meta-Analyses (PRISMA) [43]:

- Identification: 112,547 records from the Scopus database were identified, considering “all fields” for each key search term, “all document types”, and all data published in the “data range” (all years—February 2021).
- Screening: The “article title, abstract, and keywords” were designated for each term, so that 109,418 records were excluded.
- Eligibility: Of the 3129 records as document type, only the “articles” were selected to guarantee the quality of the peer review process. In this third step, 845 records were excluded.
- Included: Data were selected in the period “all years–2020”, that is, from the first article published on the subject until the last full year (2020), and only selected, to avoid a distortion of the sample, the subject areas “Arts and Humanities”, “Social Sciences”, and “Psychology”. In this fourth and final phase, 1300 documents were excluded from the 2284 records, so the final sample incorporated 984 articles (open access and non-open access).

### 3.2. Data Processing

Data processing consists of translating it into usable information. Processing must be done correctly so as to not adversely affect the findings acquired. This phase begins with raw data to convert them to a readable format, so that it is about giving them the form and context necessary for their interpretation, thus drawing adequate conclusions that allow optimal decision-making [44].

In this research, the variables studied were year of publication, journal, author, author’s country of affiliation, affiliation research institution, and keywords that determine the article.

Bibliometric studies use (i) activity indicators, which provide data on the volume and impact of research activities; and (ii) structural relationship or collaboration, which seek interactions between the items in the publications. In this study, these indicators were analyzed [45]. The quantity indicators refer to the different counts to measure the number of publications per period, in addition to the productivity of journals, authors, research institutions, and countries/territories [46,47].

To estimate the connections between the different driving agents (researchers, institutions, journals, and countries/territories) of this topic, the co-citation analysis was applied, which starts from the assumption that between two or more documents that are co-cited in a third and later, there is, from the citing author’s perspective, a thematic similarity, and that the higher the frequency of co-citation, the greater the affinity between them [48].

Moreover, to measure the relations between the keywords that define the articles, a co-occurrence analysis was applied, which allows for the identification of the current and forthcoming key research topics, since this method suggests that the articles can be reduced to the set of joint appearances between the words that compose it [49].

VOSviewer software (version 1.6.16, Center for Science and Technology Studies, Leiden University, The Netherlands) has been applied to examine the relationship indicators by the co-citation and co-occurrence method, which offer data on the interactions and the evaluation of subject-matters, which allow the activities of research networks to be measured. Therefore, this tool enables the visualization of relationship maps and network links between authors, institutions, country, journals, and keywords [50]. VOSviewer allows, within the research framework, to recognize research trends based on the use of keywords in the articles of the selected sample.

In VOSviewer terminology, the display maps include items, which are the objects of interest (publications, researchers, countries, journals, or terms). A link is a connection or a relationship between two elements. Each link has a strength, characterized by a positive numerical value, so the higher this value, the stronger the link. The items and links together constitute a network. Any two items can be grouped into a cluster, which is a set of items included in a map. Items have attributes such as the cluster number to which they have been assigned as well as the weight and score. Therefore, the weight of an item indicates its importance in the subject analyzed. A score attribute can indicate any numeric property of the elements. The scoring attribute allows elements to be compared, that is, a higher score than the other element means greater revelation in the study area; therefore, terms with a higher relevance score provide a better indication of a research topic. For this reason, determining the relevance score for each keyword assumes that the highest scoring keywords provide a better calculation to recognize a emerging line of research [51,52,53].

The results obtained are useful for the different interest groups involved in research on emotional creativity in artistic education, which require an analysis of the scientific literature for subsequent decision-making.

### 3.3. Keyword Co-Occurrence Analysis

In order to detect current and emerging lines of research on the subject of emotional creativity in art education, based on the analysis of co-occurrences of the keywords of the documents that make up the sample, the specific terminology of the VOSviewer tool was applied:

- Link: co-occurrence links between keywords.
- Total link strength: strength (positive numerical value) of each link and, for concurrent links, indicates the number of documents in which two keywords appear together.
- Cluster: set of keywords included in a network map.
- Network map: set of keywords and links.

Likewise, it is key to note that clusters do not need to comprehensively cover all components of a network map [54]. Moreover, the attributes, signified by numerical values, used to describe the keywords were “weight” and “score”. The “weight of a keyword” reveals the meaning of the keyword in the field of research studied. Hence, for a keyword, “link weight” reveals the number of links a keyword has with other terms, while “link strength weight” indicates the total strength of the links of a keyword with other keywords.

Likewise, the “score” attribute allows the keywords in the titles and abstracts of the subject papers on emotional creativity in art education to be ranked by relevance. Calculating the relevance score for each keyword assumes that the highest scoring keywords provide a better prediction to identify future research lines [50,55]. Starting with the keyword x in research field *a*, which in turn is part of research field *b*, the relevance score of keyword x in research area *a* is calculated as follows (1):

(1) $$Relevance\;score\;=\;\frac{n_{ax}}{n_{bx}+c}\;,$$

where *n_ax_* and *n_bx_* are the number of elements in the areas *a* and *b* in which the keyword *x* appears, respectively.

This mathematical relationship is based on the equilibrium of the frequency of appearance of *x* in area *a*, symbolized by the parameter *c*, in relation to the frequency of appearance of *x* in area *b*, in addition to the absolute frequency of the appearance of *x* in the area, which can be reflected as an indicator of the relevance of *x* to the area *a* [56,57].

## 4. Results and Discussion

The findings of this research are shown according to productivity, units of analysis, and temporality. These were categorized in clusters to respond to the collaboration of authors, journals, countries/territories, and research institutions, in addition to the network of keywords that describe the intrinsic meaning of the articles.

### 4.1. Evolution of Scientific Production

The temporal distribution of scientific activity is an adequate datum since it allows us to observe the flow of published documents and the importance that a research issue is acquiring.

Table 1 shows the number of articles per decade in this field of research from 1917 to 2020. The year with the highest scientific production was 2020, with 169 articles (17.17% of total). In the last decade (2011–2020), 774 articles (78.66%) were published, while the last five (2016–2020) were 532 (54.07%). Likewise, during this last decade, the highest percentage of variation (48%) occurred in the last two years analyzed, 2019 (114 articles) and 2020. These results demonstrate the interest in the study topic.

**Figure 2:**
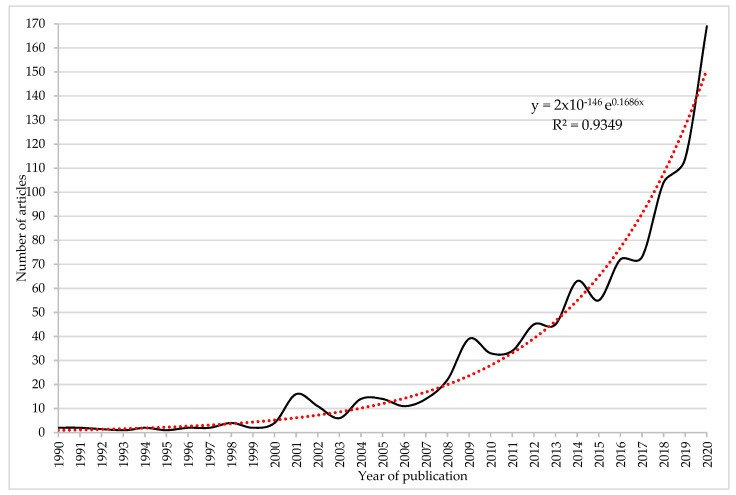
Progression of the number of published articles (1990–2020).

Figure 2 shows the evolution of scientific production on the research of emotional creativity in art education from 1990 to 2020. It is necessary to clarify that only the period 1990–2020 was selected since from 1917 to 1989, only eight articles have been published and their distortionary representation of the graphic and its trend. In this sense, the exponential trend line denotes that the number of articles on emotional creativity in art education increased more rapidly over time in the period analyzed. This line displayed its goodness of fit with a coefficient of determination (R^2^) of 0.9349, which refers to the proportion of the variance in the dependent variable (y = number of articles) that is predictable from the independent variable (x = year of publication). In the first year analyzed, 1917, only one article was published, while in the last year studied, 2020, a volume of 169 articles was published, indicating 17.17% of the total. Hence, the exponential increase in the number of articles in the analyzed period was sustained mostly by journals that had only published one article.

Initially, according to the Scopus database, the articles on the study topic were classified into 27 subject areas. Subsequently, the publications were related to the subject of “Social Sciences”, “Psychology”, and “Arts and Humanities” to limit the analysis with the variables of the study topic, that is, emotional creativity and art education [58,59].

The selected sample on this topic was written in 14 different languages. Hence, 90.96% (895) of the articles were written in English. This aspect is related to the fact that the editors consider that the publication in this language broadens the audience of the manuscript, as happens in the searches carried out in Scopus [60]. The documents had also been published in Spanish (55, 5.59%). The rest of the languages did not reach 1% of the published articles.

On the other hand, according to the Scopus database, the total number of journals that have been published on this subject was 591, that is, 159 journals (26.90%) with more than two articles and 432 (73.10%) with one article. The 10 most productive journals were: *Nurse Education Today*, with 34 articles; *Frontiers in Psychology*, with 16; *BMC Medical Education*, with 13; *Medical Education*, with 11; *Computers and Education*, *Education and Training*, and *Electronic Journal of Research in Educational Psychology*, with nine each; *Journal of Surgical Education*, with eight; and *Environmental Education Research*, *Health Psychology*, and *Medical Teacher*, with seven each.

**Figure 3:**
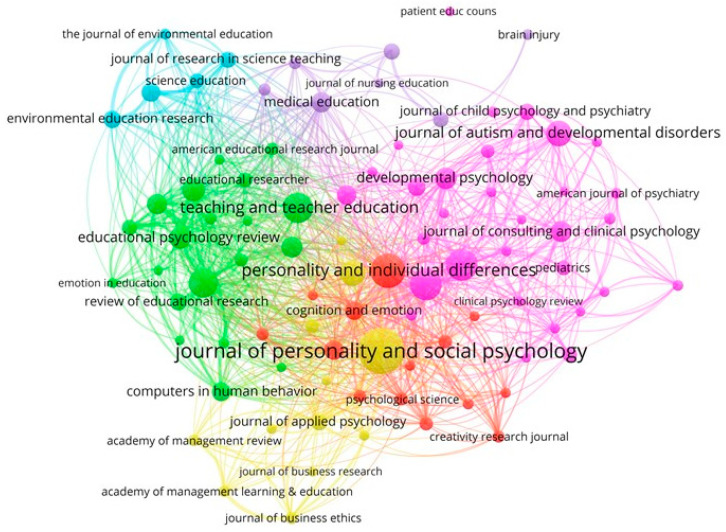
Network of journals based on the co-citation method (1917–2020).

Figure 3 shows the network map of the journals that have published internationally on emotional creativity in art education based on the co-citation method. It is necessary to indicate that in some journals, its title cannot be shown due to the high density of the cluster, thus avoiding overlap. In the visualization maps: (i) the size of the circle refers to the weight of the journal, that is, the greater the weight, the larger the circle; (ii) the color of the node indicates the cluster to which the journal belongs; (iii) the lines between the elements represent links; and (iv) the distance between two nodes reveals the relationship of the journals in terms of citation links. The journals in the period analyzed (1917–2020) were associated in six clusters, showing a high concentration. For the main journals of each cluster, it is indicated: the color that represents it, the percentage of elements that make it up over the total, the links, the total link strength, and the citations.

- Cluster 1 (pink, 35%): *Child Development* (links: 76, total link strength: 2057, citations: 184); *Psychological Bulletin* (85, 2829, 182); *Journal of Autism and Developmental Disorders* (49, 1205, 139); *Developmental Psychology* (74, 1397, 108); *Journal of Consulting and Clinical Psychology* (67, 1323, 101); *Science* (82, 1142, 95); *Plos One* (80, 729, 81); *Journal of Child Psychology and Psychiatry* (62, 1022, 71); *Pediatrics* (46, 286, 63); and *Health Psychology* (42, 541, 62).
- Cluster 2 (green, 22%): *Teaching and Teacher Education* (73, 1653, 173); *Journal of Educational Psychology* (75, 3309, 162); *Educational Psychologist* (76, 2276, 115); *Educational Psychology Review* (75, 2137, 109); *Computers & Education* (63, 1001, 104); *Learning and Instruction* (72, 1988, 99); *Computers in Human Behavior* (59, 1352, 90); *Review of Educational Research* (72, 1291, 74); *Contemporary Educational Psychology* (67, 1446, 67); and *Educational Researcher* (63, 748, 64).
- Cluster 3 (red, 16%): *Personality and Individual Differences* (86, 3062, 207); Psychological Review (77, 1374, 95); *Cognition and Emotion* (79, 1365, 86); *Personality and Social Psychology Bulletin* (76, 981, 55); *Frontiers in Psychology* (74, 896, 54); *Psychological Science* (79, 973, 53); *Creativity Research Journal* (49, 825, 49); *Emotion Review* (69, 868, 47); *Psicothema* (68, 546, 47); and *Psychology of Music* (41, 448, 41).
- Cluster 4 (yellow, 13%): *Journal of Personality and Social Psychology* (86, 4860, 310); *American Psychologist* (81, 1764, 136); *Journal of Applied Psychology* (73, 1458, 72); *Annual Review of Psychology* (77, 801, 60); *Journal of Business Ethics* (31, 944, 48); *Academy of Management Learning & Education* (32, 813, 46); *Academy of Management Review* (46, 629, 44); *Organizational Behavior and Human Decision Processes* (62, 724, 37); *Journal of Organizational Behavior* (58, 594, 36); and *Journal of Business Research* (42, 295, 34).
- Cluster 5 (purple, 8%): *Medical Education* (64, 2030, 99); *Nurse Education Today* (40, 465, 70); *Journal of Advanced Nursing* (64, 550, 67); *Academic Medicine* (53, 918, 47); *Medical Teacher* (57, 862, 47); *Brain Injury* (8, 107, 40); and *Journal of Nursing Education* (31, 256, 34).
- Cluster 6 (cyan, 6%): *International Journal of Science Education* (50, 1172, 89); *Environmental Education Research* (33, 883, 80); *Journal of Research in Science Teaching* (55, 1087, 72); *Science Education* (54, 1024, 63); and *The Journal of Environmental Education* (28, 691, 42).

The co-citation analysis made it possible to identify and recognize the dynamics of journal clusters, to analyze the strengthening and consolidation of these as well as the creation of new clusters. Thus, cluster 1 was the one with the highest density and centrality and includes the leading global journals such as *Psychological Bulletin Journal, Journal of Consulting and Clinical Psychology, Science, Nature*, or *Lancet*. Clusters 2 and 4 included peripheral and specialized journals. Cluster 3 included the second most prolific journal in the analyzed period, *Frontiers in Psychology*, and these developed relevant lines on emotional creativity in art education. Furthermore, cluster 5, which included the most cited and productive journal, *Nurse Education Today*, and Cluster 6 was composed of journals that analyzed well-developed problems.

This classification of scientific research published by the driving agents of this subject by subject, related to the criteria of centrality and density, assumes that scientific research, understood as the set of systematic and empirical processes applied to its study, is dynamic and evolutionary. For this reason, it is (i) basic, when it produces knowledge and theories such as those dedicated to the aesthetic education of emotion [61] or a conceptualization of emotion within art and design education [62,63], or (ii) applied when solving practical problems. For this reason, the dissemination of scientific research culminates when it is published in a scientific journal; since only then will it be known by the academic community, its results will be discussed, and its contribution will be part of universal scientific knowledge. In this context, the research topic on emotional creativity in art education has been attracting a growing number of journals and authors in the analyzed period, as shown by the increase in the number of published articles and the variety of interested journals globally [64,65,66].

The article that most matched the search terms in Scopus, that is, the most relevant, was published in 2020, in the journal *ARTSEDUCA. Revista electrónica de educación en las Artes* (Publisher: Universidad Jaume I de Castellon, Spain), with the title “Emotions in the artistic experience: Keys to educational and social development”, in the subject areas: Arts and Humanities (General Arts and Humanities) and Social Sciences (Education), and was written by the authors Calderón, D., Gustems-Carnicer, J., Martín-Piñol, C., Fuentes-Moreno, C., and Portela-Fontán, A. [67].

The first article was published in 1917 under the title “The education and control of the emotions”, published in the *Journal of Educational Psychology* (Publisher: American Psychological Association), in the subject areas: Social Sciences (Education) and Psychology (Developmental and Educational Psychology), and written by author Henry, T.S. [68].

The most cited article with 519 citations in February 2021 was published in 1995 by the journal *Development and Psychopathology* (Publisher: Cambridge University Press), with the title “Promoting emotional competence in school-aged children: The effects of the PATHS curriculum”, in the subject areas: Psychology (Developmental and Educational Psychology) and Medicine (Psychiatry and Mental Healthy), and was written by Greenberg, M.T., Kusche, C.A., Cook, E.T., and Quamma, J.P. [10].

### 4.2. Outputs of Driving Agents: Authors, Institutions, and Countries/Territories

The 984 articles analyzed were written by 2877 researchers worldwide. The five most prolific authors during this period were Bogner, F.X. (Universität Bayreuth, Department of Biology Education, Bayreuth, Germany), with five; Greenberg, M.T. (Pennsylvania State University, University Park, United States) and Pérez-Escoda, N. (University of Barcelona, Barcelona, Spain), with four; and Filella, G. (Universitat de Barcelona, Barcelona, Spain) and Rees, C.E. (Monash University, Melbourne, Australia), with three. Of these authors, only Bogner, F.X. published on this topic in 2020.

**Figure 4:**
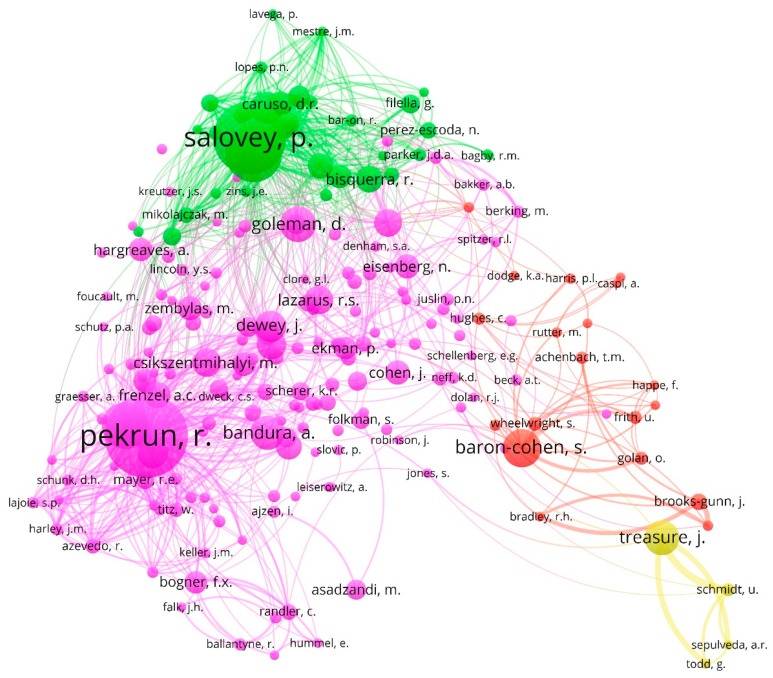
Network of authors based on co-citation method (1917–2020).

Figure 4 displays the network map based on the co-citation method among the researchers who have published on the topic between 1917 and 2020. Next, the four clusters in which the authors were associated are indicated, defined by the color in which they are represented in Figure 4 and the percentage of authors that agglutinate on the total while each author indicates the links, the total link strength, and the citations of the main authors.

- Cluster 1 (pink, 73%) Pekrun, R. (links: 158, total link strength: 7571, citations: 268); Goetz, T. (145, 4055, 132); Goleman, D. (141, 1438, 94); Bandura, A. (142, 1254, 88); and Dewey, J. (106, 495, 79)
- Cluster 2 (green, 16%): Salovey, P. (156, 7665, 227); Mayer, J.D. (151, 5362, 164); Brackett, M.A. (119, 3717, 81); Bisquerra, R. (89,1287, 67); and Furnham, A. (104, 1668, 62).
- Cluster 3 (red, 9%): Brooks-Gunn, J. (12, 1205, 37); Wheelwright, S. (52, 810, 30); Frith, U. (31, 527, 28); Golan, O. (16; 532; 27); and Achenbach, T.M. (41; 357; 26).
- Cluster 4 (yellow, 2%): Treasure, J. (38, 2666, 90); Schmidt, U. (16, 1631, 28); Sepulveda, A.R. (7, 908, 19); and Todd, G. (7, 1093, 18).

Table 2 reveals the ten most prolific research institutions on emotional creativity in the art education topic during the period 1917–2020. For each one, its publication rank is indicated, and the three main keywords associated with its contributions. It is remarkable to mention that the five most productive institutions contributed to the research topic in 2020. Keywords were mainly associated with affective and psychological states (academic emotion, assessment of emotional competence, behavior management, coexistence, cognition, communication, developmental disorder, emotion, emotional competence, emotional education, emotional intelligence, motivation, psychology, and well-being), educational aspects (academic emotion, education, and learning), and with terms of artistic content (art gallery, arts, and creativity).

The academic departments of creative arts at the world’s leading universities, because of the cutting edge in design, art and creative technologies that they anticipate, are investigating the capture and compilation of proven methods and lessons learned during the COVID-19 pandemic [69,70]. The aim will be to offer advice and practices on the experience gained in relation to the diversity and richness with which students have worked online and related psychological aspects, so that terms related to brain science occupy the first positions [71].

**Figure 5:**
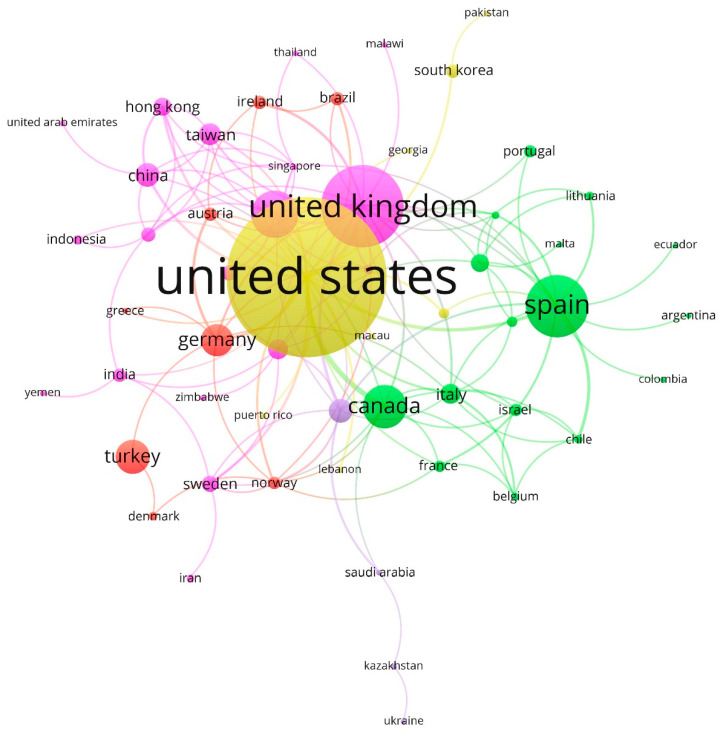
Network of countries/territories based on co-citation method (1917–2020).

Figure 5 shows the network between the leading countries/territories based on the co-citation method. These were gathered into five clusters:

- Cluster 1 (pink, 35%): the UK (links: 22, total link strength: 44, documents: 119, citations: 2494), Australia (10, 15, 59, 806), and China (8, 11, 25, 35). This group includes Taiwan (7, 9, 23, 294).
- Cluster 2 (green, 29%): Spain (16, 28, 84, 515), Canada (12, 25, 54, 720), Italy (10, 13, 21, 755), Finland (7, 7, 18, 83), and Israel (5, 5, 11, 447).
- Cluster 3 (red, 15%): Turkey (2, 2, 40, 143), Germany (8, 13, 37, 1024), Austria (4, 6, 12, 188), Brazil (3, 4, 12, 201), and Ireland (4, 6, 12, 323).
- Cluster 4 (yellow, 13%): USA (29, 67, 270, 6290), South Korea (2, 3, 12, 24), Mexico (2, 3, 9, 21), Lebanon (1, 2, 2, 7), and Pakistan (1, 1, 2, 33).
- Cluster 5 (purple, 8%): Netherlands (10, 18, 25, 209), Saudi Arabia (3, 4, 4, 11), Kazakhstan (2, 2, 3, 2), and Ukraine (1, 1, 1, 0).

The results obtained in the collaboration between countries/territories based on the co-citations method show that the U.S., the most prolific country by the number of articles published and the most cited, led cluster 4, and from the citing author’s perspective, there was a thematic similarity with articles published by authors affiliated with institutions in South Korea, Mexico, Lebanon, or Pakistan. This is due to the fact that the U.S. is a benchmark in the research of psychological and artistic studies that are related to this topic, so the countries with less production do cite the references [72,73].

Likewise, cluster 1, led by the United Kingdom, was strongly associated with Australia, China, or Taiwan. In the latter, the link with this group referred to Taiwanese policies, based on the strategy of promotion and development of artistic production, which makes efforts to develop artistic talent, enriching research for the creation and appreciation of art [74].

### 4.3. Analysis of Keywords: Lines of Research

**Figure 6:**
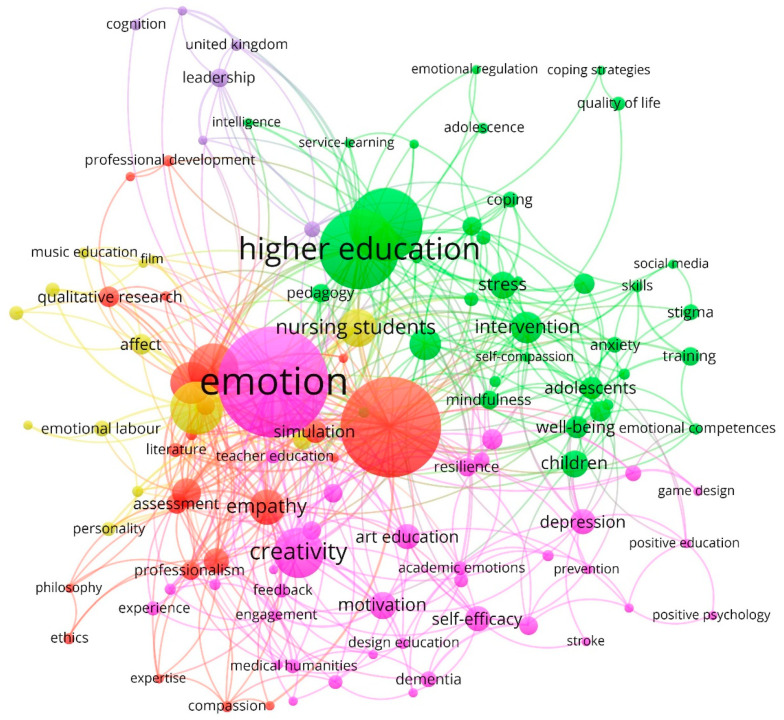
Network of keywords based on the co-occurrence method (1917–2020).

Figure 6 displays the network map for the keywords of the research articles on emotional creativity in art education during the period 1917–2020. The color of the nodes is used to differentiate the clusters based on the number of co-occurrences, while their size varies according to the number of repetitions. Furthermore, some lines of research developed by the different groups were identified. Five main lines of research were distinguished, which were grouped under the terms “Emotion”, “Higher Education”, “Education”, “Art”, and “Leadership”.

Table 3 shows the five identified clusters ordered by the weight that each group represents over the total sample. Moreover, for each cluster, the color with which each of them is displayed is indicated (see Figure 6) and the main keyword identified with an asterisk, which defines its name, and the five most prominent keywords with which it is associated within the same component. For each keyword, the weight of the occurrences, the links, and the total strength of the link are provided.

A detailed discussion of each of the identified research directions is provided below.

- **Emotion:** This line of research has highlighted emotion as a psychophysiological reaction, which represents different modes of adaptation to certain stimuli. Etymologically, the word emotion means impulse, movement, and refers to what moves a person toward something [75,76]. Relating emotion to creativity and art education refers to generating an emotional response in people, through sensory interaction, generating a more pleasant and intimate experience, creating a link that goes beyond simple action or artistic performance [77].
- **Higher Education:** The second account of knowledge has analyzed higher education as the academic level that refers to the last stage of the formal learning process of an individual [78,79]. The changes in today’s society require professionals enriched from the artistic field in their sensitivity, so that, through their own imaginary, they are creative in their contexts and from their cultures and collaborate in the development of identity in the people who make up the micro and microsocieties, in other words, that they give a sense of humanity and creativity to their professional practice [6].
- **Education:** This research approach has analyzed the process of facilitating learning or the acquisition of knowledge as well as skills, values, beliefs, and habits [80]. Art education is presented as an area of intervention aimed at the development and construction of the person-learner based on competencies acquired from the artistic culture that encourages the training of each person to develop the aesthetic and artistic sense, whether or not the student is vocationally an artist or wants to be, in the future, a professional of an art [79,81].
- **Art:** This line of research has analyzed art as a general field of education that provides common educational values linked to the character and meaning of education just like any other educational subject and as a specific development, linked to the conceptual meaning of the area of artistic experience, that is, as an area that is part of the development of the aesthetic and artistic sense [82,83].
- **Leadership:** This approach questions art education and creativity, understood as tools to increase capacities for leadership, awareness, and policy-making in terms of creative and aesthetic development [15]. Likewise, it has sought to disseminate the individual and social repercussions of art education to sensitize the public to its values and stimulate support for it in the public and private sectors [84].

### 4.4. Emerging Lines of Research

After reviewing the literature, analyzing the main drivers and keywords on emotional creativity in art education, a grouping analysis was carried out to decompose the analysis units into groups of similar elements and establish the newest terms. Hence, these key terms obtained can be considered assimilable to future thematic lines in this field of research (see Section 3.3*. Keyword Co-occurrence Analysis*). This process is a competent method for discovering emerging research topics in a scientific discipline [53]. Table 4 shows the emerging lines of research by the relevance score, and a description of each of them is added.

## 5. Conclusions

The aim was to examine the evolution of scientific research at the global level in the period 1917–2020 on emotional creativity in art education. Mathematical and statistical techniques were applied to 984 articles extracted from the Scopus database. To avoid distortions in the results, the sample only included articles from the subject areas Arts and Humanities, Social Sciences, and Psychology. International collaborations between journals, authors, research institutions, and countries/territories were studied.

Five lines of research developed from 1917 to 2020 were identified, which mainly focused on the study of: emotion, higher education, education, art, and leadership. Likewise, research worldwide on emotional creativity in art education continues to evolve, so this study has identified future directions: (i) visual art education; (ii) affective paradigm; (iii) metacompetency; (iv) expressive arts therapy group; and (v) cognitive empathy.

This study is not without limitations, which may serve as a basis for future research: (i) some leading researchers on certain topics publish few papers, but these are of great relevance; (ii) bibliometric techniques could be combined with other qualitative or quantitative methodologies to increase results and discussions; or (iii) expand the study with articles extracted from other databases.

The key contribution of this research is to generate new quantitative and qualitative knowledge, which will serve as an entry point for future discussions. On the other hand, the identification of emerging directions in research allows academics and researchers to reorient their future works, and institutions and funding centers to ensure an adequate approach to their objectives.

## Figures and Tables

**Table 1:** Number of articles per decade (2017–2020).

Period	Articles	%	Accumulated Articles	% Accumulated
2011–2020	774	78.66%	984	100.00%
2001–2010	180	18.29%	210	21.34%
1991–2000	20	2.03%	30	3.05%
1981–1990	5	0.51%	10	1.02%
1971–1980	3	0.30%	5	0.51%
…	…	...	…	…
1920–1911	2	0.20%	2	0.20%
Total	984	100.00%		

%: percentage of the total articles.

**Table 2:** Top 10 research institutions (1917–2020).

Research Institution	A	City, Country	1st A *	Last A *	Keyword 1	Keyword 2	Keyword 3
Columbia University in the City of New York	14	New York, USA	1998	2020	Emotion	Cognition	Developmental Disorder
Universitat de Barcelona	12	Barcelona, Spain	2011	2020	Emotional Competence	Emotional Education	Emotional Intelligence
Helsingin Yliopisto	12	Helsinki, Finland	2008	2020	Motivation	Academic Emotion	Emotion
King’s College London	10	London, UK	2001	2018	Emotion	Communication	Education
University of California	10	San Francisco, USA	2010	2020	Emotion	Education	Psychology
University of Valencia	9	Valencia, Spain	2011	2020	Emotion	Cognition	Well-being
The University of Manchester	8	Manchester, UK	1999	2017	Emotion	Creativity	Learning
University of Toronto	8	Toronto, Canada	2009	2019	Art Gallery	Arts	Behavior Management
The University of Edinburgh	8	Edinburgh, UK	1980	2019	Emotion	Psychology	Learning
Universitat de Lleida	8	Lleida, Spain	2008	2019	Emotional Competence	Coexistence	Assessment of Emotional Competence

A: number of articles; US: United States; UK: United Kingdom; 1st A: First article; Last A: Last article; (*) in this research topic.

**Table 3:** Clusters of keywords (1917–2020).

Cluster (Number, Color) (See Figure 6)	%	Main Keywords	Weight Links	Weight Total Link Strength	Weight Occurrences
1, pink	32%	Emotion (*)	48	74	52
		Creativity	19	29	23
		Motivation	11	15	12
		Depression	12	13	11
		Self-Efficacy	10	10	11
		Art Education	8	10	11
2, green	31%	Higher Education (*)	30	46	37
		Emotional Intelligence	23	40	34
		Intervention	21	25	14
		Emotion Regulation	18	19	14
		Stress	12	16	12
		Well-Being	13	14	10
3, red	20%	Education (*)	41	64	47
		Learning	22	34	23
		Communication	20	28	19
		Empathy	24	31	16
		Reflection	14	17	13
		Medical Education	15	18	11
4, yellow	11%	Art (*)	22	31	24
		Nursing Student	14	22	17
		Affect	11	12	9
		Emotional Labor	5	6	7
		Personality	5	9	6
		Design	4	4	6
5, purple	6%	Leadership (*)	7	9	8
		Gender	11	12	7
		Emotional Competence	6	6	5
		Cognition	2	2	5
		Decision Making	5	7	4
		Professional Education	5	6	4

(*) Main keyword that gives name to the cluster.

**Table 4:** Future research directions.

Future Direction of Research	Relevance Score	Main Associated Terms	Description
Visual ArtEducation	38.593	Artistic Fencing Craft Art Art School	It seeks to promote through the various artistic disciplines, transmit, and create more complex elements with visual, expressive, and aesthetic characteristics, both traditional and the newest and unconventional trends offered by new technologies such as digital art, urban art and other more emerging in the last decades [78,85].
Affective Paradigm	25.451	Affective Domain Cognitive Disability Self Determination	This paradigm attempts to create new ways of investigating emotions from the perspective of art and the social sciences. It is sought that emotions and affections gain strength; and also, interactions, discourses, the body or gender (and its cultural and historical variability), as social and psychic mobilizers; and other powerful knowledge builders [86,87].
Metacompetency	23.263	Verbal Creativity Positive Education Reliability	This line of research should analyze the internal, spiritual, psychic, and emotional competencies that support the competencies of doing, and without which that doing lacks quality and meaning. This context refers to the underlying values and principles that support the being and doing of a person and their very reason for being [88,89]. For this reason, critical thinking and knowledge in art favors the development of metacompetencies while criticism is an inherent condition of art, of the work of art and aesthetics. The critical or self-critical character toward systems of representation is a fundamental principle of contemporary art and is what allows a work of art to be cataloged and differentiated from any utilitarian object, therefore, the interest in its knowledge and development [37].
Expressive Arts Therapy Group	12.171	Art Therapist Cognitive Therapy Social Safety	Expressive arts therapy, also known as creative therapies, refers to the use of artistic media as a form of therapy [9]. Unlike traditional art expression, it places its emphasis on the process of creation and not on aesthetic value or the final product. The expressive arts are a powerful way to provoke questions, communicate knowledge, inspire understanding, and transform society [90]. This process invites people, groups, and communities to participate in a certain creative space where, through different artistic modalities, they express, identify, and enhance their emotional resources, in order to strengthen their ability to imagine and transform their feelings into a positive [91,92].
Cognitive Empathy	11.193	Discrete Emotion Positive Influence Achievement Emotion	Empathy seeks to understand and recognize what the other person is feeling, but always from the intellect, never from one’s own emotion [93]. By relating art education to empathy, results can be obtained in terms of recognizing what another person thinks and vibrating even with what he or she feels. In this sense, this direction will try to analyze how to express and expand the inner world to those around us [94,95].

## Data Availability

The data were obtained from Elsevier’s Scopus database (https://www.scopus.com/) accessed on 27 February 2021.
